# Reasons for Receiving or Not Receiving HPV Vaccination in Primary Schoolgirls in Tanzania: A Case Control Study

**DOI:** 10.1371/journal.pone.0045231

**Published:** 2012-10-24

**Authors:** Deborah Watson-Jones, Keith Tomlin, Pieter Remes, Kathy Baisley, Riziki Ponsiano, Selephina Soteli, Silvia de Sanjosé, John Changalucha, Saidi Kapiga, Richard J. Hayes

**Affiliations:** 1 London School of Hygiene and Tropical Medicine, London, United Kingdom; 2 Mwanza Intervention Trials Unit, National Institute for Medical Research, Mwanza, Tanzania; 3 Medical Research Council Social & Public Health Sciences Unit, Glasgow, United Kingdom; 4 Unit of Infections and Cancer, Cancer Epidemiology Research Programme, l'Institut d'Investigació Biomèdica de Bellvitge, Institut Català d'Oncologia, Barcelona, Spain; 5 CIBER Epidemiologia, y Salud Publica, Barcelona, Spain; 6 The National Institute for Medical Research, Mwanza, Tanzania; Yale School of Public Health, United States of America

## Abstract

**Background:**

There are few data on factors influencing human papillomavirus (HPV) vaccination uptake in sub-Saharan Africa. We examined the characteristics of receivers and non-receivers of HPV vaccination in Tanzania and identified reasons for not receiving the vaccine.

**Methods:**

We conducted a case control study of HPV vaccine receivers and non-receivers within a phase IV cluster-randomised trial of HPV vaccination in 134 primary schools in Tanzania. Girls who failed to receive vaccine (pupil cases) and their parents/guardians (adult cases) and girls who received dose 1 (pupil controls) of the quadrivalent vaccine (Gardasil™) and their parents/guardians (adult controls) were enrolled from 39 schools in a 1∶1 ratio and interviewed about cervical cancer, HPV vaccine knowledge and reasons why they might have received or not received the vaccine. Conditional logistic regression was used to determine factors independently associated with not receiving HPV vaccine.

**Results:**

We interviewed 159 pupil/adult cases and 245 pupil/adult controls. Adult-factors independently associated with a daughter being a case were older age, owning fewer household items, not attending a school meeting about HPV vaccine, and not knowing anyone with cancer. Pupil-factors for being a case included having a non-positive opinion about the school de-worming programme, poor knowledge about the location of the cervix, and not knowing that a vaccine could prevent cervical cancer. Reasons for actively refusing vaccination included concerns about side effects and infertility. Most adult and pupil cases reported that they would accept the HPV vaccine if it were offered again (97% and 93% respectively).

**Conclusions:**

Sensitisation messages, especially targeted at older and poorer parents, knowledge retention and parent meetings are critical for vaccine acceptance in Tanzania. Vaccine side effects and fertility concerns should be addressed prior to a national vaccination program. Parents and pupils who initially decline vaccination should be given an opportunity to reconsider their decision.

## Introduction

Infection with human papillomavirus (HPV) is the primary cause of cervical cancer, with approximately 70% of cases caused by HPV genotypes 16 and 18 [1], [2]. Tanzania has one of the highest rates of cervical cancer and mortality from cervical cancer in the world [3]. Two HPV vaccines offer a new opportunity for primary prevention. The vaccines protect against HPV type 16 and 18 infections and associated cervical pre-cancerous lesions and, in the case of the quadrivalent vaccine, also against HPV 6 and 11, the main cause of genital warts [4], [5], [6]. Vaccination is typically targeted at young adolescent or pre-adolescent girls before they can acquire HPV after sexual debut [7]. This is not an age group that is routinely targeted by vaccination programmes in developing countries. Parental and community acceptability of a vaccine that prevents a sexually transmitted infection and how the vaccine is promoted and delivered by health-care providers will influence its uptake and vaccine effectiveness [8], [9], [10], [11]. To inform policy makers on the best delivery strategies and sensitisation messages needed when a new vaccination programme is commenced, it is important to understand factors that influence the decision to receive or not receive the vaccine

As part of a project to demonstrate the feasibility, acceptability and costs of delivering HPV vaccine in primary schools in Tanzania, we examined the characteristics of receivers and non-receivers of HPV vaccination and reasons for receiving or not receiving the vaccine.

## Materials and Methods

### Ethical considerations

Ethical approval was provided by the ethical committees of the Medical Research Coordinating Committee, Tanzania, and the London School of Hygiene and Tropical Medicine.

### HPV vaccination project activities

A phase IV cluster-randomised trial (NCT01173900) was conducted in Tanzania to compare two different vaccine delivery strategies in primary schools; age-based delivery, where the quadrivalent HPV vaccine, Gardasil™, was offered to all girls in the school who were born in 1998, and a class-based strategy where girls who were enrolled in primary school class 6 in 2010 were offered vaccination [12]. The trial was conducted between August 2010 and June 2011 and was located in Mwanza city and in ten administrative units (wards) of neighbouring Misungwi district.

In total 134 schools were randomly selected from 241 primary schools; 67 were randomised to the age-based strategy and 67 to the school-based strategy.

Three vaccine doses were offered to eligible girls during four rounds of school visits over 11 months. Dose 1 was offered over two rounds, with girls who missed dose 1 the first time being offered another opportunity to receive this during the second round of vaccination. If girls missed a dose at school, the vaccine was made available at the health facility for a period of two to four weeks after the school visit but was not left at the health facility permanently because of cold storage space limitations. Teachers were provided with a list of pupils who had missed their booked dose and asked to encourage them to attend the health facility.

Social mobilisation was conducted through parent-teacher meetings, letters to parents, meetings with ward and other community leaders and religious leaders, distribution of project leaflets, radio broadcasts and performances by community dance and drama troupes. The project adopted an opt-out consent approach for parents following consultation with key stakeholders. When information about the date of vaccination in schools was provided to parents through letters delivered by their daughters, they were asked to indicate to teachers or the project team if they did not wish their daughter to be vaccinated or to keep them back from school.

### Selection of cases and controls

To determine factors associated with not being vaccinated, we conducted a case control study on a sample of 250 girls who did not receive dose 1 (cases) and 250 girls who received dose 1 (controls). For each of the 250 cases and 250 controls, we aimed to interview both the girl and her parent/guardian. Non-receivers were girls who were eligible for vaccination but did not receive dose 1, either because they were absent from school or were reported by the teacher to be ill or their parents or the girl indicated that they did not wish to be vaccinated or the girl absconded from school on the vaccination day. Eligible girls who wished to be vaccinated but did not receive dose 1 because of suspected pregnancy, or because the study team judged them to be too unwell, were excluded from the case-control study.

Cases and controls were matched on school. In each of the selected schools (described below), we invited for interview all girls who did not receive dose 1 (either at school or at the health facility) and who were eligible to be included as cases, and an equal number of randomly-selected girls who received dose 1 as controls. For each school, a list of replacement control pupils was drawn up in the event that a control pupil or parent refused to participate in the interviews. No replacements were possible for cases within each school, since all non-receivers were invited.

### Sample size and power

To achieve the target sample size of approximately 250 parent-child cases and 250 parent-child controls, we selected a random sample of 2 private schools, 15 government urban schools and 22 government rural schools, from those schools where there was at least one potential case and one potential control (Table 1). We aimed to interview 7 parent-child cases in the private schools, and 123 parent-child cases each from the government urban and rural schools. Schools in which all eligible girls were vaccinated (N = 27) were excluded from the case-control study since there were no cases at these schools. Similarly, 4 schools in which all eligible girls failed to be vaccinated were excluded. The decision to include 39 schools was based on the number of schools needed to obtain approximately 250 cases if all non-receivers were invited to take part in each of these schools.

**Table 1:** Selection, participation and analysis of cases and controls.

	Govt rural schools (N = 22)		Govt urban schools (N = 15)		Private schools (N = 2)		All schools (N = 39)	
	Cases	Controls	Cases	Controls	Cases	Controls	Cases	Controls
**Initially selected**	**123**	**125**	**122**	**122**	**7**	**7**	**252**	**254**
**Girls**								
Interviewed from initial selection	73	109	83	96	3	6	159	211
Interviewed from replacement list	-	14	-	25	-	1	-	40
Dropped because no adult questionnaire	0	1	0	0	0	0	0	1
Dropped because no cases interviewed at school	-	2	-	2	-	1	-	5
**Adults**								
Interviewed from initial selection	81	109	84	96	3	6	168	211
Interviewed from replacement list	-	14	-	25	-	1	-	40
Dropped because no girl questionnaire	8	1	1	0	0	0	9	1
Dropped because no cases interviewed at school	-	2	-	2	-	1	-	5
**Total parent-girl pairs analysed**	**73**	**120**	**83**	**119**	**3**	**6**	**159**	**245**
*Age-based*	42	67	8	15	3	6	53	88
*Standard (class) based*	31	53	75	104	0	0	106	157

The study was powered to provide ≥80% power to detect an odds ratio (OR) of 1.75 for risk factors associated with not receiving the vaccine, assuming that the prevalence of the risk factor in the controls was between 25–65%, or 90% power for an OR of 2.00, assuming that the prevalence of the risk factor was between 20–70%.

### Enrolment and interview procedures

An interviewer visited the girl's household to ask for written or witnessed oral (if illiterate) parental/guardian consent for an interview with the parent/guardian and a separate face-to-face interview with their daughter/ward. Written informed assent of the eligible girl was sought separately once parental consent had been given.

Using pre-tested structured questionnaires, the interviewer collected quantitative data on sociodemographic information, health-seeking behaviour, especially in relation to vaccinations, knowledge about cervical cancer, and the HPV vaccine. Closed and open-ended questions on reasons for receiving or not receiving vaccination were asked at the end of the questionnaire and, apart from these, all other questions were identical for cases and controls. The child was interviewed separately from the participating adult. Some open-ended questions were also asked about satisfaction with the original decision to receive or not receive vaccination.

### Statistical considerations

Data were double-entered in OpenClinica 3.0.1 (2009; Akaza Research; Waltham, Massachusetts, USA) and analyzed in STATA 11.0 (StataCorp LP; College Station, Texas, USA).

We estimated odds ratios (OR) and 95% confidence intervals (CI) for factors associated with not receiving vaccine, using conditional logistic regression to account for clustering within schools. Potential determinants of not being vaccinated were examined using a conceptual framework with three levels: socio-economic, health-seeking behaviour, and knowledge of the HPV vaccine project. Parents' age was included in all models a priori. Socio-economic factors that were associated with not receiving vaccine at p<0.10 were included in a multivariable model and those independently associated at p<0.10 were retained in a core model. Health-seeking factors were added to this core model one by one. Those associated with not receiving vaccine at p<0.10, after adjusting for socio-economic factors, were included in a multivariable model and retained if they remained associated at p<0.10. Associations with knowledge of the HPV vaccine project were determined in a similar way. The final model excluded factors one at a time until all remaining factors were associated at the p<0.05 level.

Girls who did not receive the vaccine comprised two potentially different groups: those who were absent on the day of vaccination, but who may have wanted the vaccine, and those who attended but actively refused vaccination. We did not collect data on reasons for absence, so we could not distinguish between absence to avoid vaccination and absence for other reasons. However, we did an additional analysis to compare the characteristics of girls who were absent with those who refused vaccination, using a Pearson chi-squared test with the second-order correction of Rao and Scott to account for the clustered design.

## Results

### Vaccine coverage

Vaccine coverage results have been described previously [12], [13]. In summary, 4684/5532 (84.7%) of eligible girls enrolled in the 134 schools received dose 1; 86.4% in standard-based schools compared with 82% in age-based schools (p = 0.30).

### Enrolment and interviewees

From 252 eligible cases in the 39 randomly selected schools, we were able to locate, enrol and interview 159 (63.1%) girls (“pupil cases”) and 168 (66.7%) parents/guardians (“adult cases”, Table 1). Nine girls whose parents consented were not interviewed: 5 had moved to an unknown address, 1 was at boarding school, the parent withdrew consent for one girl, and 2 had a disability which precluded the interview. Analysis of cases was restricted to the 159 pupil/adult case pairs in 35 schools.

From the 254 randomly selected controls in the same 39 schools, we located, enrolled and interviewed 211 (83.1%) pupils (“pupil controls”) and 211 (83.1%) parents/guardians (“adult controls”). Of these, one adult control did not have a matching pupil interview because the girl had moved away, while one pupil control did not have matching adult data because the parent questionnaire could not be located. A further 40 adult/pupil control pairs were enrolled from a list of replacement controls. Five pupil/adult control pairs from four schools were not included in the analysis because there were no matching cases at those schools. Analysis of controls was restricted to the remaining 245 pupil controls and adult controls in 35 schools.

Over half of the adults interviewed were mothers (Table 2). The median age of adult interviewees was 40 (IQR 34–49) for adult cases and 37 (IQR 32–45) for adult controls; 67% of adult cases and 77% of adult controls were married. No education was reported by 16% of adult cases and 9% of adult controls. Only 13% of cases and 17% of controls owned five or more of listed household items (radio, cell-phone, television, bicycle, motorbike, car, livestock or a plot of land); 6% of adult cases and 1% of adult controls owned none of these items.

**Table 2:** Univariate analysis of factors associated with girls not receiving HPV vaccine: characteristics of parents/guardians.

	Cases (N = 159)		Controls (N = 245)		Unadjusted OR [95% CI]^1^
	N	*%*	N	*%*	
**SOCIO-DEMOGRAPHIC FACTORS**					
**Relationship to girl**					P = 0.58
Mother	90	*56.6*	*131*	*53.5*	1
Father	28	*17.6*	*36*	*14.7*	1.05 [0.59, 1.86]
Other female relative	32	*20.1*	*62*	*25.3*	0.73 [0.44, 1.22]
Other male relative	9	*5.7*	*16*	*6.5*	0.74 [0.31, 1.77]
**Age (years)**					P = 0.08
<30	12	*7.5*	31	*12.6*	1
30–39	64	*40.3*	112	*45.7*	1.60 [0.76, 3.38]
40–49	42	*26.4*	63	*25.7*	1.80 [0.82, 3.97]
50+	35	*22.0*	32	*13.1*	3.12 [1.35, 7.23]
Age not known	6	*3.8*	7	*2.9*	2.46 [0.64, 9.45]
**Highest level of education**					P = 0.09
Secondary/higher	15	*9.4*	33	*13.5*	1
Primary	119	*74.9*	189	*77.1*	1.34 [0.68, 2.62]
None	25	*15.7*	23	*9.4*	2.44 [1.03, 5.79]
**Marital status**					P = 0.006
Married	107	*67.3*	189	*77.1*	1
Divorced/separated/widowed	43	*27.0*	37	*15.1*	2.22 [1.33, 3.71]
Single	9	*5.7*	19	*7.8*	0.85 [0.36, 1.98]
**Occupation (highest in household)**					P = 0.25
At least one professional/business	63	*39.6*	113	*46.1*	1
No professional/business	96	*60.4*	132	*53.9*	1.30 [0.83, 2.04]
**Religion**					P = 0.32
Christian	129	*81.1*	206	*84.1*	1
Other/none	30	*18.9*	39	*15.9*	1.33 [0.76, 2.33]
**Number of items owned** 2					P = 0.004
5 or more	21	*13.2*	42	*17.2*	1
2–4	110	*69.2*	174	*71.0*	1.49 [0.82,2.72]
1	19	*11.9*	27	*11.0*	1.62 [0.71,3.70]
0	9	*5.7*	2	*0.8*	15.98 [2.92,87.55]
**Number of children**					P = 0.16
1–3	38	*23.9*	79	*32.2*	1
4–6	78	*49.1*	112	*45.7*	1.52 [0.94, 2.47]
> 6	43	*27.0*	54	*22.1*	1.62 [0.90, 2.91]
**HEALTH SEEKING BEHAVIOUR**					
**Girl treated by de-worming programme**					P = 0.06
Yes	79	*49.7*	146	*59.6*	1
No	80	*50.3*	99	*40.4*	1.47 [0.98, 2.24]
**Adult's opinion of de-worming programme**					P = 0.03
Good	101	*63.5*	183	*74.7*	1
Did not like/no opinion	58	*36.5*	62	*25.3*	1.64 [1.06, 2.54]
**Adult's trust in government health institutions**					P = 0.55
Trust	148	*93.1*	225	*91.8*	1
Little/no trust/don't know	11	*6.9*	20	*8.2*	0.79 [0.37, 1.71]
**Girl immunised at MCH clinic**					P = 0.79
Yes – all immunisations	125	*78.6*	199	*81.2*	1
Yes – some immunisations	17	*10.7*	22	*9.0*	1.27 [0.64, 2.53]
No immunizations/don't know	17	*10.7*	24	*9.8*	1.08 [0.55, 2.13]
**Adult immunised in childhood**					P = 0.008
Yes	103	*64.8*	190	*77.6*	1
No	56	*35.2*	55	*22.4*	1.82 [1.17, 2.85]
**KNOWLEDGE AND ATTITUDES**					
**Adult's first awareness of HPV vaccine**					P = 0.08
Through project source	50	*31.4*	95	*38.8*	1
Through non-project source	109	*68.6*	150	*61.2*	1.49 [0.95, 2.32]
**Adult attended meeting to discuss vaccine**					P = 0.002
Yes	36	*22.6*	88	*35.9*	1
No	123	*77.4*	157	*64.1*	2.11 [1.32, 3.39]
**Adult known anyone made ill/died from cancer**					P<0.001
Yes	79	*49.7*	163	*66.5*	1
No	80	*50.3*	82	*33.5*	2.17 [1.40, 3.36]

1 Odds ratios are calculated from conditional logistic regression (conditioned on school).

2 Possible items owned are : radio; cellphone; television; bicycle; motorcycle; car; live-stock; agricultural plot.

Not all adults were aware of whether their respective daughters/wards had received the vaccine. Of the 159 adult cases, 109 (68%) believed that the pupil had not received the vaccine, 9 (6%) thought that she had received it, and 41 (26%) did not know. Of the 245 adult controls, 196 (80%) believed that the pupil had received the vaccine, 14 (6%) thought that she had not received it, and 35 (14%) did not know.

The median age of interviewed pupils was 13 years (IQR 13–15 years) for cases and 13 years (IQR 13–15) for controls. Overall 19% of cases and 10% of controls reported frequently being absent from school (Table 3).

**Table 3:** Univariate analysis of factors associated with girls not receiving HPV vaccine: characteristics of girls.

	Cases (N = 159)		Controls (N = 245)		Unadjusted OR [95% CI]^1^
	N	*%*	N	*%*	
**SOCIO-DEMOGRAPHIC FACTORS**					
**Age of girl (years)(N = 263)** 2					P = 0.02
≤12	6	*5.7*	18	*11.5*	1
13–14	66	*62.3*	72	*45.9*	3.10 [1.12, 8.59]
15–16	22	*20.7*	53	*33.8*	1.47 [0.48, 4.52]
≥17	12	*11.3*	14	*8.9*	3.37 [0.91, 12.41]
**Girl's class (standard)(N = 141)** 3					P = 0.54
Class 3	5	*9.4*	10	*11.4*	1
Class 4	8	*15.1*	17	*19.3*	1.21 [0.25, 5.90]
Class 5	19	*35.9*	38	*43.2*	1.23 [0.30, 5.14]
Class 6	21	*39.6*	23	*26.1*	2.14 [0.48, 9.61]
**Girl ever absent from school**					P = 0.04
Never	66	*41.5*	110	*44.9*	1
Occasionally	63	*39.6*	110	*44.9*	1.02 [0.64, 1.61]
Sometimes/frequently	30	*18.9*	25	*10.2*	2.19 [1.15, 4.18]
**HEALTH SEEKING BEHAVIOUR**					
**Girl received other vaccinations at school**					P = 0.08
Yes	40	*25.2*	45	*18.4*	1
No	119	*74.8*	200	*81.6*	0.64 [0.39, 1.05]
**Girl treated by de-worming programme**					P = 0.002
Yes	93	*58.5*	176	*71.8*	1
No	66	*41.5*	69	*28.2*	2.00 [1.29, 3.12]
**Girl's opinion of de-worming programme**					P<0.001
Good	91	*57.2*	185	*75.5*	1
No opinion/did not like	68	*42.8*	60	*24.5*	2.60 [1,66, 4.08]
**Girl's source of treatment when ill**					P = 0.79
Government clinic/hospital	114	*71.7*	173	*70.6*	1
Private clinic/hospital/pharmacy	45	*28.3*	72	*29.4*	0.94 [0.59, 1.50]
**KNOWLEDGE AND ATTITUDES**					
**Girl's first awareness of HPV vaccine**					P<0.001
Through project source	140	*88.1*	243	*99.2*	1
Through non-project source	19	*11.9*	2	*0.8*	17.61 [4.00, 77.62]
**Girl attended meeting to discuss vaccine**					P = 0.001
Yes	42	*26.4*	99	*40.4*	1
No	117	*73.6*	146	*59.6*	2.05 [1.31, 3.21]
**Girl known anyone with cervical cancer**					P = 0.74
Yes	3	*1.9*	5	*2.0*	1
No	156	*98.1*	240	*98.0*	1.27 [0.30, 5.48]
**Girl's knowledge of location of cervix**					P<0.001
Top of vagina	16	*10.1*	64	*26.1*	1
In uterus/reproductive system	28	*17.6*	62	*25.3*	2.03 [0.96, 4.30]
Abdomen/another part/don't know	115	*72.3*	119	*48.6*	3.99 [2.10, 7.58]
**Girl's knowledge of cause of cervical cancer**					P = 0.01
Correct/partially correct answer	8	*5.0*	*30*	*12.2*	1
Incorrect answer	151	*95.0*	*215*	*87.8*	2.59 [1.15, 5.85]
**Girl's knowledge of cervical cancer prevention**					P<0.001
Vaccine specifically mentioned	84	*52.8*	182	*74.3*	1
No mention of vaccine, but any of screening or condom use or no sex	9	*5.7*	10	*4.1*	2.12 [0.82, 5.49]
Other, not including any of those above	66	*41.5*	53	*21.6*	2.67 [1.69, 4.23]
**Girl ever had sexual intercourse**					P = 0.14
Yes	11	*6.9*	10	*4.1*	1
No	148	*93.1*	235	*95.9*	0.50 [0.20, 1.26]

1 Odds ratios are calculated from conditional logistic regression.

2 Girl's age is based on schools with standard-based vaccination strategy only.

3 Girl's standard is based on schools with age-based vaccination strategy only.

### Factors associated with not receiving the vaccine

From project and teacher records, 85 (53%) of 159 cases did not receive dose 1 of vaccine because the pupil was absent from school on the vaccination day, 70 (44%) because a parent refused, 2 (1%) because the girl refused and 2 (1%) for other reasons. Amongst cases, more parents in the “absent from school” group had received no education compared with the “school, attender/refuser” group (20% vs. 8% respectively; p = 0.003) and there was a higher proportion of parent cases in the “absent” group who heard about the vaccine through a non-project source than in the “attender/refuser” group (78% vs. 57%; p = 0.008). Pupils in the “absent” group were older than in the “refuser” group (28% aged 15–16 vs. 6%, respectively; p = 0.004). There were no other significant differences between these groups.

In the unadjusted analysis, adult-reported factors associated with the pupil being a case included being divorced, separated or widowed, owning fewer household items, not having attended a teacher-parent meeting to discuss the cervical cancer vaccine, having a non-positive opinion of the school de-worming programme and not knowing anyone who had had cancer (Table 2). Increasing age, lower education, hearing about the vaccine from a non-project source, the pupil not being treated by the de-worming programme, and the adult not being immunised in childhood were weakly associated with the pupil not receiving HPV vaccine. Having little or no trust in government health institutions was not significantly associated with being unvaccinated.

Pupil-reported factors associated with being a case in the unadjusted analysis included age, ever being absent from school, first hearing about the HPV vaccine from a non-project source, not attending a school meeting to discuss the vaccine, not having been treated by the de-worming programme, not having a positive opinion (i.e. no opinion or did not like) of the de-worming programme, not knowing the location of the cervix in the body, not knowing the cause of cervical cancer and not mentioning vaccine as a method to prevent cervical cancer (Table 3). Not having received other vaccinations (e.g. tetanus toxoid) at school had a borderline association with being a case. Having passed sexual debut was not associated with being unvaccinated. On multivariable analysis (Table 4), adult factors that were independently associated with the pupil being unvaccinated were increasing age (adjusted OR (aOR) 3.62; 95%CI 1.39–9.58 for those ≥50 years compared with those <30 years), owning fewer household items (aOR 12.71; 95%CI 2.11–76.75 for not owing any of selected household items), not attending a school meeting about the HPV vaccine project (aOR 2.31; 95%CI 1.34–3.98) and not knowing anyone with cancer (aOR 2.12; 95%CI 1.28–3.49). Pupil-reported factors that were independently associated with not having received the vaccine were having a non-positive opinion about the school de-worming programme (aOR 1.92; 95%CI 1.14–3.23), poor knowledge about location of the cervix (aOR 2.65; 95%CI 1.17–6.01 for somewhere in reproductive system; aOR 3.37; 95%CI 1.62–6.99 for not known or somewhere in the abdomen or elsewhere in the body), and not knowing that a vaccine could prevent cervical cancer (aOR 2,73; 95%CI 0.91–8.13 for mentioning screening, condoms or no sex; aOR 1.78; 95%CI 1.04–3.06 for not known or methods excluding vaccination, screening, condoms or no sex).

**Table 4:** Multivariate analysis of factors independently associated with girl not receiving HPV vaccine.

	Adjusted OR [95% CI]^1^
**Age of adult interviewee (years)**	P = 0.03
<30	1
30–39	1.32 [0.57, 3.09]
40–49	1.97 [0.81, 4.79]
50+	3.62 [1.37, 9.58]
Age not known	2.16 [0.45, 10.48]
**Number of items owned by adult**	P = 0.02
5 or more	1
2–4	1.47 [0.73, 2.94]
1	2.11 [0.81, 5.45]
0	12.71 [2.11, 76.75]
**Adult known of anyone made ill by/died from cancer**	P = 0.003
Yes	1
No	2.12 [1.28, 3.49]
**Adult attended teacher/parent meeting to discuss vaccine**	P = 0.002
Yes	1
No	2.31 [1.34, 3.98]
**Girl's opinion of deworming programme**	P = 0.01
Good	1
No opinion/did not like	1.92 [1.14, 3.23]
**Girl's knowledge of location of cervix**	P = 0.003
Top of vagina	1
In uterus/reproductive system	2.65 [1.17, 6.01]
Abdomen/another part/don't know	3.37 [1.62, 6.99]
**Girl's knowledge of cervical cancer prevention**	P = 0.03
Vaccine specifically mentioned	1
No mention of vaccine, but any of screening or using condoms or no sex	2.73 [0.91, 8.13]
Other, not including any of those above	1.78 [1.04, 3.06]

1 Exposures are adjusted for all other potential risk-factors in the model. Odds ratios are calculated from conditional logistic regression.

### Adult and pupil reasons for receiving or not receiving vaccination

Cases and controls were asked why they had or had not received vaccination. Multiple answers were allowed. Although controls and cases were selected from vaccine records, 58 (20%) adult controls stated that they thought their daughters had not received vaccine. Among the 196 adult controls who reported that their daughter had received vaccine, their reasons for accepting vaccination (Table 5) included protection against cervical cancer (N = 175; 89%), health benefits (N = 43; 22%), knowing someone who had had cancer (N = 25; 13%) and encouragement by the project team (N = 19; 10%). Only 6 (3%) were not happy with their decision, citing a lack of consultation/information and concern over side effects.

**Table 5:** Parent/guardian and pupil satisfaction with decision to accept or refuse HPV vaccination.

Controls	N (%)	Cases	N (%)
**Parent/guardians** 1	196	**Parent/guardians** 2	109
**Happy with decision to vaccinate**	190 (96.9)	**Happy with decision not to vaccinate**	32 (29.4)
*Reasons for satisfaction with decision*		*Reasons for satisfaction with decision*	
Will provide protection against cervical cancer	55 (28.9)	Concern over side effects	8 (25.0)
Will provide protection against cancer	19 (10.0)	Concern over infertility	8 (25.0)
Will provide general protection	99 (52.1)	Unspecified worries	7 (21.9)
Vaccine safety/absence of side-effects	6 (3.2)	Other reason	9 (28.1)
Other reason	11 (5.8)		
**Unhappy with decision to vaccinate**	6 (3.1)	**Unhappy with decision not to vaccinate**	77 (70.6)
*Reasons for dissatisfaction with decision*		*Reasons for dissatisfaction with decision*	
Lack of consultation/awareness	3 (50.0)	Girl missed having protection of vaccine	52 (67.5)
Concern over side-effects	2 (3.3)	Did not understand value of vaccine/insufficient information	17 (22.1)
Other reason	1 (16.7)	Other/no reason	8 (10.4)
**Pupils**	245	**Pupils** 3	153
**Happy with decision to be vaccinated**	244 (99.6)	**Happy with decision not to be vaccinated**	39 (25.6)
*Reasons for satisfaction with decision*		*Reasons for satisfaction with decision*	
Protection against cervical cancer	159 (65.1)	Avoided side effects of vaccine	7 (17.9)
Protection against disease	51 (20.9)	Fear of injections	7 (17.9)
General benefit	28 (11.5)	Fear of infertility/reproductive problems	3 (7.7)
No reason	6 (2.5)	Prevented by/obeyed parent(s)	10 (25.6)
		Other/no reason	12 (30.8)
**Unhappy with decision not to be vaccinated**	1 (0.4)	**Unhappy with decision not to vaccinate**	114 (74.4)
*Reasons for dissatisfaction with decision*		*Reasons for dissatisfaction with decision*	
Felt pressurized to be vaccinated	1 (100)	Missed protection offered by vaccination	102 (89.5)
		Obeyed parents/felt mislead	9 (7.9)
		Other/no reason	3 (2.6)

1 196/245 (80%) adult controls who reported during interviews that daughter/ward had received vaccine.

2 109 of 159 (69%) adult cases who reported during interview that daughter/ward had not received vaccine.

3 153 of 159 (96%) pupil cases who reported during interview that they had not received vaccine.

Reasons for being vaccinated, as reported by 224 (91%) of 245 pupil controls, included protection from cervical cancer (N = 225; 91%), health benefits (N = 57; 24%), and parental wishes (N = 52; 21%; Table 5). Only one girl was unhappy with the decision to be vaccinated, explaining that she felt under pressure to receive the vaccine. It was not clear whether this pressure came from parents, teachers, fellow pupils or the vaccine team.

Of the 159 adult cases, 109 (69%) reported that their daughters were not vaccinated. Reasons for not agreeing to vaccination included concern over either side effects (N = 44; 40%) or infertility (N = 22; 23%), or insufficient knowledge about the vaccine (N = 24; 22%; Table 5). Twenty-three (21%) had wanted their daughter/ward to receive the vaccine but the girl had been absent from school on the vaccination day. Overall 77 (71%) regretted their decision, of whom 52 (68) stated that the girl had missed receiving the protection conferred by vaccination, and 17 (22%) that they had not understood the value of the vaccine. Most (N = 75; 97%) said they would agree to the vaccination if it were offered again because the vaccine would protect their daughters/wards from cervical cancer (N = 60; 80%), and that the vaccine was safe (N = 43; 57%).

Overall 153/159 (96%) pupil cases reported that they had not been vaccinated, of whom 50 (33%) stated that they had been absent from school on the vaccination day, 37 (24%) that both parents had refused permission for the vaccination, 34 (22%) had concerns about side effects, 25 (16%) were afraid of injections, and 20 (13%) had infertility concerns (Table 5). In total 114 (75%) girls were unhappy about not receiving the vaccine, 102 (89%) feeling they had missed an opportunity to protect their health and 9 (8%) stating they had been forbidden by their parents to get vaccinated or were misled about side-effects. Of these 106 (93%) said that they would accept vaccination if given another opportunity to have this because it offered protection against cancer (N = 85; 80%), the vaccine was safe (N = 38; 36%), their friends had had it (N = 16; 15%) or that their parents would like them to receive it (N = 10; 9%).

## Discussion

This is the first case control study exploring individual-level factors associated with not receiving HPV vaccination in a developing country. HPV vaccination in the first HPV vaccine delivery project in Tanzania had high acceptance, with over 80% of girls receiving at least one dose of vaccine [12], [13]. With the announcement of Tanzania's plans to launch a national HPV vaccination programme in 2012–2013, it is essential to explore factors associated with receipt or non-receipt of vaccine.

Our main findings suggest that several important programmatic factors will be critical to reassure parents/guardians that HPV vaccine is safe and effective. These include parental attendance at school meetings and ensuring that girls have attended education and information sessions and retain messages about cervical cancer and the vaccine. Girls whose parents/guardians did not attend a school sensitisation meeting or girls who could not name basic factors related to the cervix and to HPV vaccine as a prevention method for cervical cancer were at high risk of not being vaccinated. Sensitisation messages will, however, need to be tailored to the target population. One study in the US has shown that provision of information through leaflets improved knowledge but was not sufficient to ensure pre-vaccination parental acceptability and concluded that attitudes and life experiences were more influential in determining HPV vaccine acceptance [11]. Other studies in the US found that recommendations from doctors influenced actual acceptance of HPV vaccine [14], [15]. In our setting, face-to-face meetings with teachers and health workers which allowed questions and concerns to be answered, and government endorsement of the vaccine as well as knowing someone with cancer probably all contributed to actual parental acceptance [16].

Parents/guardians from poorer households with few modern or desired traditional material possessions had a high risk of not having their daughters vaccinated. Poverty has been associated with poorer completion rates of HPV vaccination in the US [17]. Reasons for this are unknown but may be related to poor education, a lack of understanding of the benefits of health interventions within the household and/or poor health seeking behaviours or a preference for “traditional” forms of health care.

Older adult interviewees were also associated with poor vaccine uptake. Older household members may not have been the parents of eligible girls and may therefore have been unable to make a decision to recommend vaccination or may have had poorer health prevention behaviours or were less educated or required the pupils to help at home. Sensitisation messages will need to be specifically developed to reach older and poorer parents/guardians, through community and religious leader engagement in addition to standard approaches, and opportunities to answer their questions will need to be provided prior to the start of vaccination.

One potential life-experience that may have influenced vaccine uptake concerned the national de-worming programme. Adverse press coverage about reactions to praziquantel in Tanzania's de-worming programme in the mid-2000s led to public confusion and concern about school-based health interventions, especially vaccination, and impacted on the delivery of these programmes [18]. Public memory of such events may be long, as demonstrated by the loss in public confidence of oral poliovirus vaccine in Nigeria and the measles-mumps-rubella (MMR) vaccine in the United Kingdom [19], [20]. In our study, girls who reported a non-positive opinion about the national de-worming programmes in schools were at high risk of failing to be vaccinated. Lack of trust in this school health programme may have a significant impact on the ultimate uptake of the HPV vaccine in schools unless this concern is specifically addressed. Targeted messages should be incorporated into sensitisation information to inform and reassure parents and girls about both the benefits and safety of the de-worming and the HPV vaccination programmes.

Reassuringly, no parents/guardians of cases raised the issue that, because HPV vaccine was a vaccine against a sexually transmitted disease, this would give girls a licence to have sex. This concern has been raised by parents in some studies in developed countries [15], [21], [22] although was not universally raised in other studies [9], [16].

Multiple studies have reported on the potential acceptability of and barriers to HPV vaccination [11], [14], [21]–[29]. However intention to accept an intervention does not necessarily translate to actual acceptance. Strengths of our study include the fact that we examined factors associated with failure to receive vaccine during a vaccination programme, rather than a pre-intervention assessment. We found some similarities to results from a study in Scotland where girls were interviewed at the end of the three dose course and reported concerns about vaccine safety and efficacy as reasons for not being vaccinated [30]. Fear of side effects was an important reason for non-uptake of vaccine in our study and has been cited as a barrier to vaccination in a number of countries [29], [31]–[33]. This can substantially impact programmatic delivery and uptake of vaccine, especially following potentially misleading media coverage [34], [35].

Prevention of cervical cancer was the primary reason given for accepting the vaccine in both adults and girls. This is important since research prior to vaccination in this population had shown little or no knowledge about cervical cancer [16]. Interestingly although peer-approval has been associated with vaccination in the US [36], this was not cited as a reason for accepting vaccination in our study.

Our study shows that it will be important to give parents and pupils time to reconsider their decisions when a new national vaccination programme commences. Most adults and pupils who did not accept vaccination initially would have accepted this for their daughters/themselves if they had been offered another opportunity to do so. This could be achieved in practice by offering several visits within a school year to receive dose 1, making people aware during social mobilisation that it is acceptable to change one's mind about not accepting HPV vaccination and then encouraging girls to inform the teachers or health workers if they or their parents have reconsidered their original decision not to receive vaccine.

Study limitations include potential selection bias. Only 60% of cases participated in the study compared with more than 80% of controls and therefore the interviewed cases may not be representative of all vaccine non-receivers. However, since most cases who did not participate were people who could no longer be located, rather than actual refusals, the cases we did interview should be representative of those girls who were still attending the school but did not receive the HPV vaccine. We were not able to separate out those who were absent because they stayed away to avoid vaccination and those who absent for other reasons, and these is evidence that these two groups are potentially different since we did find some differences in parental education, how they had learnt about HPV vaccine and in pupil age. Finally, adult interviewees may not have been the primary decision maker in the household at the time of vaccination and so may have guessed the reasons behind the decision to receive or not receive vaccination and all interviewees may have experienced recall bias since the case-control study was done nearly a year after the first dose of vaccine and they may not have recalled their original reasons for refusing/accepting the vaccine.

In summary, sensitisation messages, retention of this knowledge and parent meetings are critical for vaccine acceptance. Persistent concerns about vaccine side effects and potential impact on fertility will need to be closely addressed in a national vaccination programme and steps will need to be taken to allow parents and pupils who initially decline vaccination to reconsider their decision.
